# Are You Still With Me? Continuous Engagement Assessment From a Robot's Point of View

**DOI:** 10.3389/frobt.2020.00116

**Published:** 2020-09-16

**Authors:** Francesco Del Duchetto, Paul Baxter, Marc Hanheide

**Affiliations:** Lincoln Centre for Autonomous Systems, School of Computer Science, University of Lincoln, Lincoln, United Kingdom

**Keywords:** user engagement, machine learning, tools for HRI, service robotics, robot autonomy

## Abstract

Continuously measuring the engagement of users with a robot in a Human-Robot Interaction (HRI) setting paves the way toward *in-situ* reinforcement learning, improve metrics of interaction quality, and can guide interaction design and behavior optimization. However, engagement is often considered very multi-faceted and difficult to capture in a workable and generic computational model that can serve as an overall measure of engagement. Building upon the intuitive ways humans successfully can assess situation for a degree of engagement when they see it, we propose a novel regression model (utilizing CNN and LSTM networks) enabling robots to compute a single scalar engagement during interactions with humans from standard video streams, obtained from the point of view of an interacting robot. The model is based on a long-term dataset from an autonomous tour guide robot deployed in a public museum, with continuous annotation of a numeric engagement assessment by three independent coders. We show that this model not only can predict engagement very well in our own application domain but show its successful transfer to an entirely different dataset (with different tasks, environment, camera, robot and people). The trained model and the software is available to the HRI community, at https://github.com/LCAS/engagement_detector, as a tool to measure engagement in a variety of settings.

## 1. Introduction

One of the key challenges for long-term interaction in human-robot interaction (HRI) is to maintain user engagement, and, in particular, to make a robot aware of the level of engagement humans display as part of an interactive act. With engagement being an inherently internal mental state of the human(s) interacting with the robot, robots (and observing humans for that matter) have to resort to the analysis of external cues (vision, speech, audio).

In the research program that informed the aims of this paper, we are working to close the loop between the user perception of the robot as well as their engagement with it, and our robot's behavior during real-world interactions, i.e., to improve the robot's planning and action over time using the responses of the interacting humans. The estimation of users' *engagement* is hence considered an important step in the direction of automatic assessment of the robot's behaviors in terms of its social and communicative abilities, in order to facilitate *in-situ* adaptation and learning. In the context of reinforcement learning, a scalar measure of engagement can directly be interpreted as a *reinforcement signal* that can eventually be used to govern the learning of suitable actions in the robot's operational situation and environment. As a guiding principle (and indeed a working hypothesis), we anticipate that higher and sustained engagement with a robot can be interpreted as a positive reinforcement of the robot's action, allowing it to improve its behavior in the long term.

Previous work on robot deployment in museum contexts (Del Duchetto et al., 2019) provide evidence on how user engagement during robot-guided tours easily degrades with time when employing an open-loop interactive behavior that does not take into account the engagement state of the other (human) parties.

However, we argue that the usefulness of a scalar measure of engagement as presented in the paper stretches far beyond our primary aim to use it to guide learning. Work in many application domains of HRI (Baxter et al., 2014; Ben-Youssef et al., 2017; Rudovic et al., 2017) has focused on a measure of engagement to inform the assessment of the implementation for a specific use-case or to guide a robot's behavior. However, how engagement is measured and represented varies greatly (see section 2) and there is yet to be found a generally applicable measure of engagement that readily lends itself to guide the online selection of appropriate behavior, learning, adaptation, and analysis. Based on the observation that engagement as a concept is implicitly often quite intuitive for humans to assess, but inherently difficult to formalize into a simple and universal computational model, we propose to employ a data-driven machine learning approach, to exploit the implicit awareness of humans in assessing an interaction situation. Consequently, instead of aiming to comprehensively model and describe engagement as a multi-factored analysis, we use end-to-end machine learning to directly learn a regression model from video frames onto a scalar in the range of 0 to 100%, and use a rich annotated dataset obtained from a long-term deployment of a robot tour guide in a museum to train said model.

For a scalar engagement measure to be useful in actual HRI scenarios, we postulate that a few requirements have to be fulfilled. In particular, the proposed solution should

- Demonstrably generalize to new unseen people, environments, and situations;
- Operate from a robot's point of view, forgoing any additional sensors in the environment;
- Employ a sensing modality that is readily available on a variety of robot platforms;
- Have few additional software dependencies to maximize community uptake; and
- Operate with modest computational resources at soft real-time.

**Figure 1:**
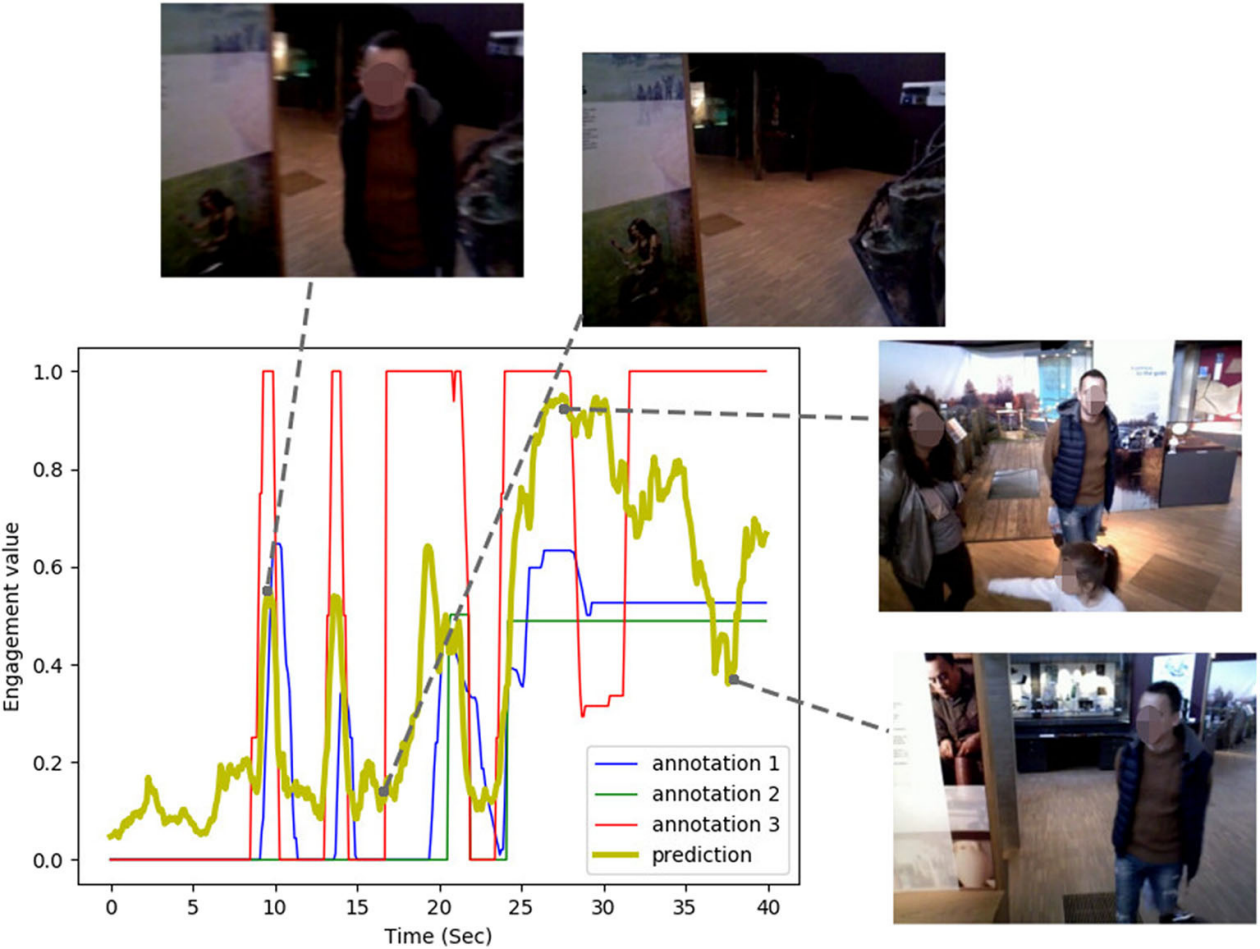
Engagement annotated values and our model's predictions over a guided tour interaction sequence recorded from our robot's head camera. Faces from original dataset blurred for anonymisation.

Consequently, we present our novel engagement model, solely operating on first-person (robot-centric) point of view video of a robot and prove its applicability not only in our own scenario but also on a publicly available dataset (UE-HRI) without any transfer learning or adaptation necessary. Figure 1 shows our model's predictions for a brief video sequence of interactions from our dataset and how it compares with the ground truth annotations. We demonstrate that the model can operate at a 5 Hz frame rate on average GPU hardware typically found on robots. Hence, the core contributions of this paper can be summarized as

1. The appraisal of a scalar engagement score for the purpose of *in-situ* learning, adaptation, and behavior generation in HRI;
2. A proposed end-to-end deep learning architecture for the regression of first-person view video stream onto scalar engagement factors in real-time;
3. The comprehensive assessment of the proposed model on our own long-term dataset, and a publicly available HRI dataset proving the generalizing capabilities of the learned model; and
4. The availability of implementation and trained model to provide the community with an easy to use, out of the box methodology to quantify engagement from the first-person view video of an interactive robot.

## 2. Assessment of Engagement

Recognizing the level of engagement of humans during the interactions is an important capability for social robots. In the first place, we want to recognize the level of engagement as a way to assess the robot behavior. Feeding this information to a learning system we can improve the robot behavior to maximize the level of engagement. In an education scenario, such as a museum, being able to engage the users is a crucial factor. It is known that a higher level of engagement generates better learning outcomes (Ponitz et al., 2009), while engagement with a robot during a learning activity has also been shown to have a similar effect (Gleason and Greenhow, 2017). While there is evidence that the presence of a robot, particularly when novel, is sufficient in itself for higher engagement in educational STEM activities, e.g., Baxter et al., 2018, the focus in the present work is on engagement between individuals and the robot within a direct (social) interaction, for which there is not a universally agreed definition (Glas and Pelachaud, 2015).

### 2.1. Definitions of Engagement

Regarding the conceptual definition of engagement, in the attempt to define *what* engagement is, the literature has taken two different approaches: the first, that views engagement as a process that takes place during the interactions and, the second, that defines it as a metric of the interaction quality, which value can be estimated from observations. Within the first group of works (Sidner et al., 2004, 2005) describe engagement as “the process by which individuals in an interaction start, maintain and end their perceived connection to one another” and “it combines verbal communication and non-verbal behaviors, all of which support the perception of connectedness between interactors.” O'Brien and Toms (2008) define engagement with technology as “a process comprised of four distinct stages: point of engagement, period of sustained engagement, disengagement, and reengagement”; with the process being characterized “by the presence of multiple attributes that vary in intensity depending on a combination of user and system attributes that emerge during the interaction”. The attributes considered are “challenge, aesthetic and sensory appeal, feedback, novelty, interactivity, perceived control and time, awareness, motivation, interest, and affect.”

Between the works that have taken the assumption that engagement is a metric of the interaction, we find (Peters et al., 2005) which defines engagement as “the value that a participant in an interaction attributes to the goal of being together with the other participant(s) and of continuing the interaction”. They propose that it is relevant to assess engagement during two different moments of the interaction: at the moment of starting a communicative interaction (to assess the possibility of engagement in interaction) and when the interaction is going on (to check if engagement is lasting and sustaining conversation). According to (Salam and Chetouani, 2015b) the engagement is a social dimension that can be seen as “the measure of the intention-to and the quality-of interaction as perceived by the user.”

In our work, we pursue the latter idea, i.e., that engagement is a measure of the interaction quality that can be evaluated during social interactions, rather than attempting to detect the different phases that compose the engagement process.

### 2.2. Characterization of Engagement

Peters et al. (2005) identifies engagement and interest as causal factors of attention and, therefore, devise an algorithm based on gaze for detecting engagement in interactions. Additionally to interest and attention, an affective component (e.g., valence) can be integrated into the characterization of engagement from the perception of the user's facial features (Castellano et al., 2014) and from the robot's own affective expressions (Castellano et al., 2009). In both human-human and human-robot interactions human gaze has been identified as of particular significance when determining engagement levels in an interaction, e.g., Rich et al., 2010; Holroyd, 2011. Gaze thus forms an important behavioral cue when assessing engagement, e.g., Sidner et al., 2004; Baxter et al., 2014. For example, Lemaignan et al. (2016) do not try to directly define and detect engagement, recognizing that it is a complex and broad concept. Instead, the concept of “with-me-ness” is introduced, which is the extent to which the human is “with” the robot during the interactions, and which is based on the human gaze behavior.

Beyond only non-verbal behaviors (Foster et al., 2017), attempt at estimating the engagement state of customers from the audio-visual sensors data of a robot bartender. Sidner et al. (2004, 2005) also combines verbal communication (user utterances and sound location) with non-verbal behaviors, to “support the perception of connectedness between interactors.”

Context has also been identified as being of importance, in terms of the task and environment, as well as the social context (Castellano et al., 2012). For example, Michalowski et al. (2006) proposes a simple model to infer engagement for a robot receptionist based on the person spatial position within some predefined areas around the robot, and Salam and Chetouani (2015a) attempts to predict the engagement of one entity in a multiparty interaction relying only on the features of rest of the group, showing that engagement, and the features needed to detect it, changes with the context of the interaction (Salam and Chetouani, 2015b). Similar results from Leite et al. (2015) show that the prediction of disengagement in a one-person interaction vs. a multi-party interaction relies on different features. These examples furthermore suggest that there are multiple, overlapping, and likely interacting timescales involved in the characterization of engagement, from the longer-term context to short interaction-orientated behaviors that nevertheless impact social dynamics, and which humans are particularly receptive to Durantin et al. (2017).

In addition to these explicitly cue-centered approaches, more recently, attempts have been made to leverage the power of machine learning to discover the important overtly visible features with minimal (or at least sparse) explicit guidance from humans (through cue identification for example). For example, Won Park et al. (2019) uses an active learning approach with Deep RL to automatically (and interactively) learn the engagement level of children interacting with a robot from raw video sequences. The learning is incremental and allows for the real-time update of the estimates so that the results can be adapted to different users or situations. The DQN is initially trained with videos labeled with engagement values. In other work, Rudovic et al. (2018) investigate the performance of a deep learning model, called CultureNet, to specifically estimate the engagement of children with autism coming from different cultural backgrounds and study the performance across the multicultural data, although this is based on a dataset of images of the children's faces rather than real-time data.

These deep learning methods have the advantage that the constituent features of interest do not have to be explicitly defined *a priori* by the system designer, rather, only the (hidden) phenomenon needs to be annotated; engagement in this case. Since social engagement within interactions is readily recognized by humans based on visible information (see discussion above), human coding of engagement provides a promising source of ground-truth information. Indeed, in this context, Tanaka et al. (2007) employed human coders to assess the “quality” of observed interactions, demonstrating good agreement between coders on what was a subjective metric.

Taken together, the literature indicates that while a precise operational definition of engagement may not be universally agreed, it seems that more holistic perspectives may be more insightful. It is likely that while gaze is an important cue involved in making this assessment, there are other contextual factors that influence the interpretation of engagement. Given that humans are naturally able to accurately assess engagement in interactions, it seems that one promising possibility would be to leverage this to directly inform automated systems.

## 3. Preliminaries

**Figure 2:**
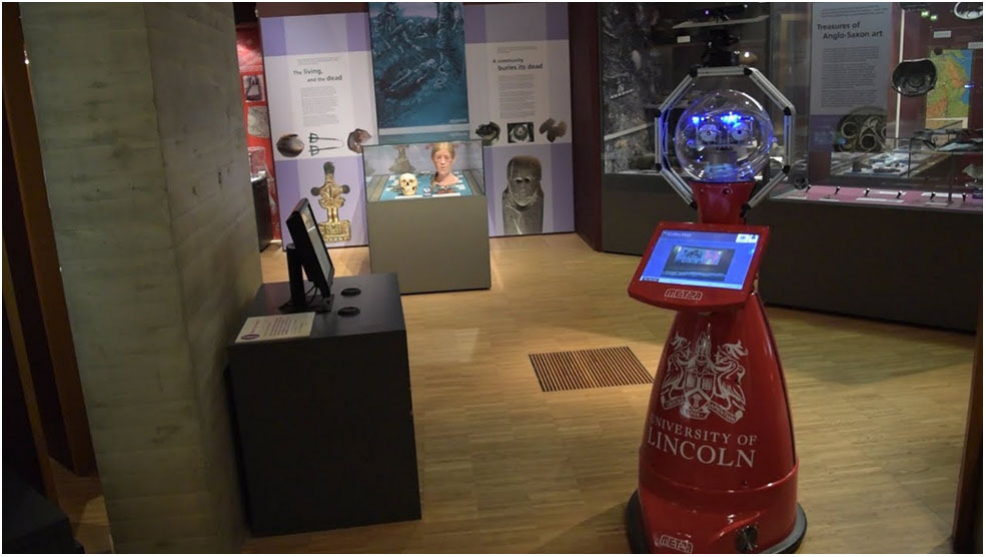
Lindsey, the tour guide robot deployed at The Collection museum.

This work is embedded in a research program that seeks to employ online learning and adaptation of an autonomous mobile robot to deliver tours at The Collection museum in Lincoln, UK^1^. The robotic platform, described below and shown in Figure 2, has been operating autonomously in this environment for an extended period of time, with the goal to facilitate the visitor's engagement with the museum's display of art and archaeology. This project provides an opportunity to study methodologies to equip the robot with the ability to interact socially with the visitors. In particular, the research aims to find a good model to allow the robot to do the correct thing at the right moment, in terms of social interaction. The first step in doing so is endowing the robot with a means of assessing its own performance at any given moment to allow adaptation, learning, and to avoid repeating the same errors.

In this section, we briefly report the architecture of the robot system, a description of the robot behaviors and an up-to-date analysis of the deployment. For a more detailed description of these aspects of the project, the reader is redirected to our previous work (Del Duchetto et al., 2019).

### 3.1. Robotic Platform

The robot is a Scitos G5 robot manufactured by MetraLabs GmbH. It is equipped with a laser scanner with 270° scan angle on its base and two depth cameras. An Asus xtion depth camera is mounted on a pan-tilt unit above his head and a Realsense D415 is mounted above the touchscreen with an angle of 50° with respect to the horizontal plane in order to face the people standing in front of the robot. The interactions with the visitors are mediated through a touch screen, two speakers, a microphone array and a head with two eyes that can move with five degrees of freedom to provide human-like expressions. To ensure safe operations in public environments the robot is equipped with an array of bumpers around the circular base with sensors to detect collisions and two easily reachable emergency buttons that, when activated, cuts the power to the motors. The software framework is based on ROS and uses the STRANDS project (Hawes et al., 2017) core modules for topological navigation, people tracking, task scheduling, and data collection.

### 3.2. Robot Behaviors

During the deployment, the robot actively tries to engage with those people that are detected in its surrounding by gazing toward them and saying “Hi there! Do you want some information about this museum? Interact with my screen interface!”. If the users approach the robot, they can interact through the robot's touchscreen to browse the museum map and to start one of the tasks that the robot makes available. During the execution of such tasks, every information is pronounced verbally by the robot and replicated with text on the upper part of the screen. The users can communicate verbally with the robot only in specific moments of the tasks to answer yes/no questions from the robot. In parts of the task the robot also shows images, related to the exhibits it is showing, and information about the task in execution (e.g., where the robot is navigating to or if the robot is listening to the user utterances). We describe below the three different tasks that the users can initiate with the robot.

#### 3.2.1. Guided Tour

Each tour is centered around a theme (chosen by the user) and is made of a predefined set of exhibits to be traversed in the same order all the time. The robot initially gives a description of the tour providing some context for the exhibits. Then it guides the visitors through the stops of the tour sequentially, for each giving some brief information and successively asking the visitors if they want to know more. The visitors can reply with the touchscreen through a yes/no modal window or by verbally pronouncing their answer. The robot guides the visitors to the next stop in case of a negative answer.

#### 3.2.2. Go to Exhibit and Describe

The robot guides the visitors to an exhibit of their choice and then describes it, with a short description initially and, optionally, a more detailed one.

#### 3.2.3. Describe Exhibit

The robot gives a short verbal description of the exhibit demanded by the visitor.

### 3.3. Long Term Deployment Analysis

**Figure 3:**
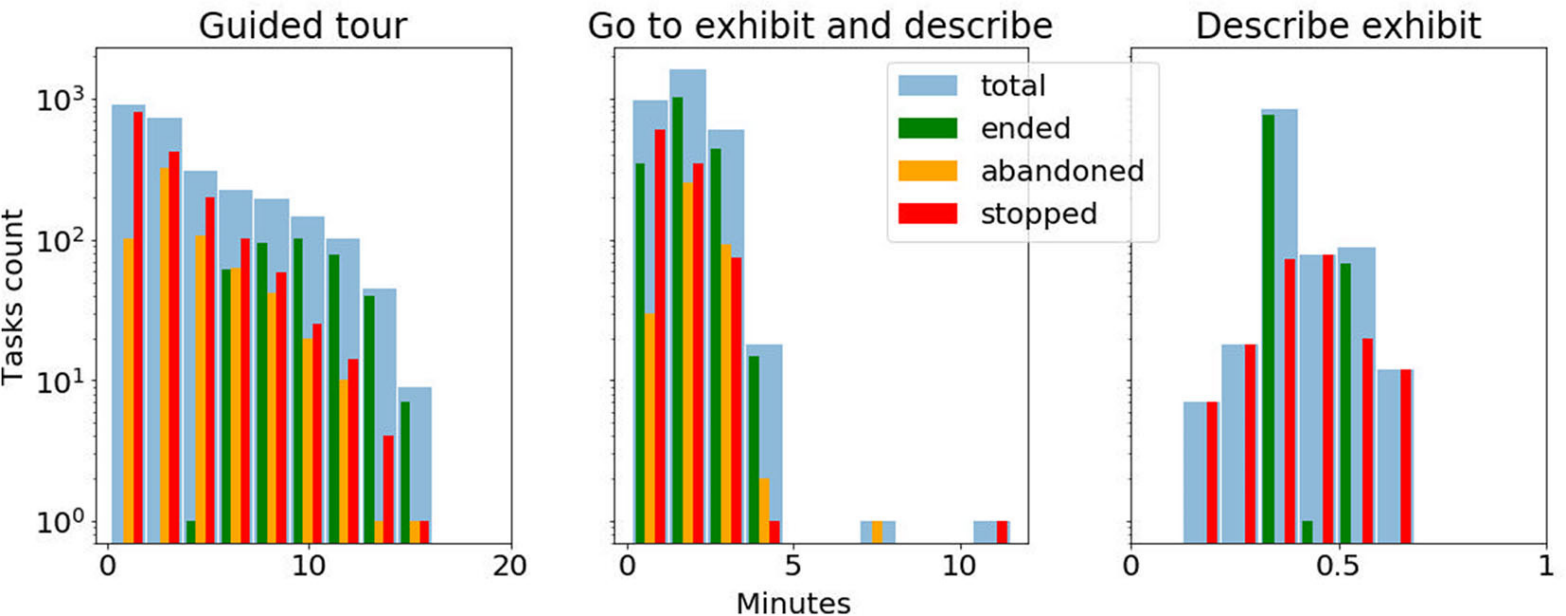
Duration distribution (in log scale) of the interactive tasks performed by the robot. The different colors green, orange and red represent respectively the tasks that were completed to the end, were abandoned by the users or were stopped by the users. The light blue bars sums up the three task groups.

The robot's deployment at the museum has started in October 2018 and is continuing to date, with minimal periods of interruption for robot maintenance or the museum being closed. In total the robot has been operative, and available to the public for interactions, for 278 days and has traveled 556 km in the museum. In order to analyse the number of interactions with the users, we report the amount and duration for each task category in Table 1. In the analysis we filter out those tasks during which one of the following failures occurred: (1) the robot localization accuracy is low, (2) some areas of the museum are blocked for the robot to navigate into, (3) the robot's emergency stop button is pressed, (4) the robot's bumper detects a collision, or (5) system failure. Whenever the robot is executing a task, the user has the ability to stop it by pressing a button on the robot's touchscreen. The event is detected by the robot system which stops the task. The users can go away from the interaction while a task is executing without explicitly stopping it, in which case the task is classified as abandoned. The robot classifies a task as abandoned whenever it does not receive an answer by 1 min time after asking a question to the users. Figure 3 reports the duration distribution of the tasks comparing those that ended normally with those tasks that were either stopped or abandoned. For abandoned tasks, the duration includes the 1 min of waiting for the user's feedback. This data taken together evidences the long term autonomy abilities of the robot in the current deployment, but also the need for a mechanism to assess and increase the users' engagement during the interactions.

**Table 1:** Number of user demanded tasks with their duration.

**Task**	**Tot. demanded**	**Average duration**	**Shortest**	**Longest**
*Guided tour*	2691 (5365)	4 (4.4) [min]	11 (11) [s]	16.1 (23.4) [min]
*Go to exhibit and describe*	3246 (4824)	1.7 (1.8)[min]	8.4 (7.5) [s]	11.5 (30.8) [min]
*Describe exhibit*	1048 (1111)	21.6 (24) [s]	7.3 (7.3) [s]	41 [s] (5.8 [min])

*Between parenthesis are reported the values obtained before filtering out the tasks in which a failure has occurred*.

Data collection has been in place during almost the entire period of the deployment, in particular, we collected data about the robot internal state and from its sensors during the interactions with the museum visitors. The work and data recording exercise have been approved by the University of Lincoln's Ethics Board, under approval ID “COSREC509.” The ethical approval does not allow the public release of any data that can feature identifiable persons, in particular video data. The data utilized in the analysis reported in this section span the date range between the 24th January 2019 (the day on which we started recording data of the robot operations) and the 17th March 2020, with data collection remaining ongoing.

## 4. The TOGURO Dataset

### 4.1. Dataset Collection

The TOur GUide RObot (TOGURO) dataset was collected from the two cameras mounted on the robot's body and head, each providing a stream of RGB and depth frames. Each video stream is recorder from the moment the user starts a *guided tour* or a *go to exhibit and describe* task until its termination, therefore it does not include the initiation of engagement phase. We have excluded from the dataset collection the *describe exhibit* tasks considering their generally short duration time. Considering the large number of videos to be stored, each frame coming from the cameras is collected, compressed and stored as MPEG videos while the interaction is taking place. To be able to reconstruct the frame-by-frame alignment between the different video streams, we store, parallelly to the frames, the ROS timestamp at the time each frame is received by the video recorder node.

The participants were aware that the robot was recording data during the interactions (by means of visible signs and leaflets), although they were not informed that the purpose of this data was for engagement analysis, thus not biasing their behaviors. In total, we collected 3,106 distinct videos for a total duration of about 10 days and 16 h of recorded interactions for each camera stream. Note, however, that only a small subset of this total data was coded and used for training/evaluating the proposed model, as described below (section 4.2).

Given that the museum in which the robot is deployed is a public space openly accessible to anyone, the interactions between the robot and the museum's visitors are completely unstructured. People walking in the gallery are allowed to roam around the collection or to interact with the robot. When they choose to do so they do not receive any instruction about how to interact with it explicitly and are not observed by experimenters when doing so.

### 4.2. Dataset Coding

In order to address the primary research goal—the assessment of robot-centric group engagement—the dataset was manually coded in order to establish a ground truth. As noted previously, given that there is not a universally accepted operationalized definition of engagement, a human observer response method is employed in the present work, following the prior application of a continuous audience response method (Tanaka et al., 2007).

Three annotators took part in the coding process: each was familiar with the robot being used and the interaction context. The annotators are students at the University of Lincoln, knew each other before the study and were not remunerated for the activity. They were instructed to provide engagement scores as scalar values in the range [0, 1] to reflect the following measure: “*Situate yourself as the guide (i.e., the robot) carrying out the interaction and looking through the cameras. How much (do you feel) the people are being engaged by the interaction with you?”*. A set of exemplary cases was also provided:

- Whenever the person is looking at the screen or at the robot head: engagement is HIGH;
- When the person is looking at the exhibit (the exhibit is typically behind the robot): engagement is HIGH;
- When the person is attending the tour but annoyed, continuously looking around, or looking at the phone: the engagement is MEDIUM;
- When the person is not attending the tour (e.g., far from the robot, oriented with the back toward the robot, talking to other people): engagement is LOW;
- When the person is not in the camera field of view: engagement is LOW;
- When the face of the people are not completely visible, do not immediately classify engagement as if the people were outside the FOV but try to guess their engagement value;

where HIGH, MEDIUM, and LOW do not identify a precise discrete value but they are an indicator of the scalar value range. Reflecting on the nature of the interactions in the museum, the score provided by the coders takes into account the situations where a user diverts its attention from the robot but remains essentially engaged in the task by looking at the exhibits.

**Figure 4:**
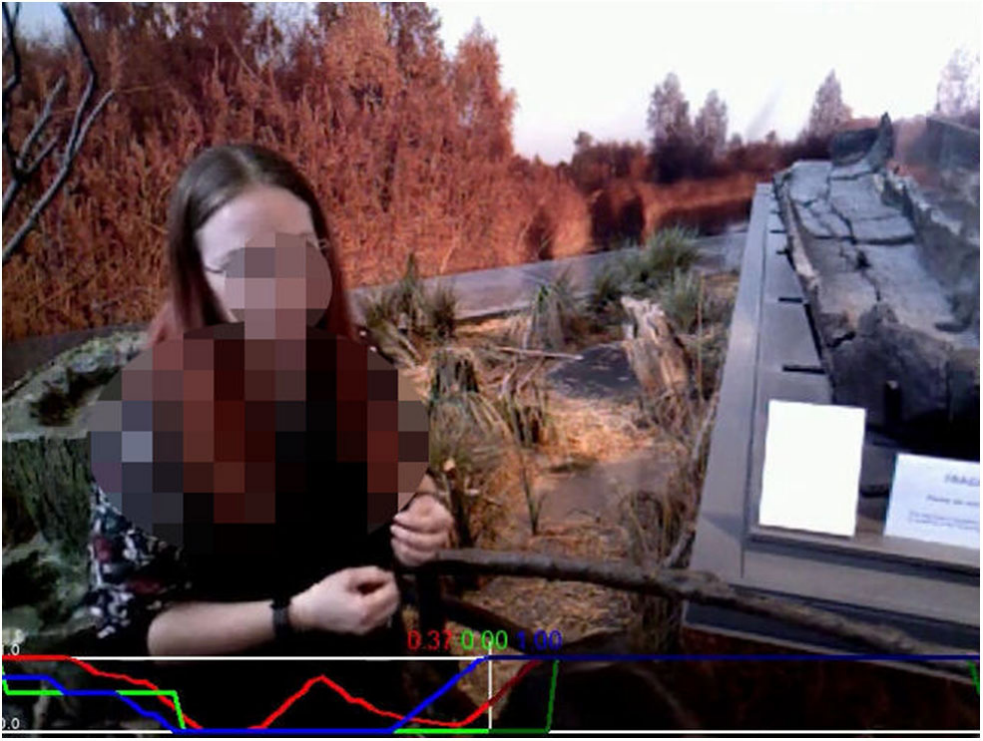
One frame from a video in the TOGURO dataset recorded from the robot's head camera during a guided tour. The red, green, and blue plots at the bottom of the frame represent each a distinct annotation sequence. Face from original dataset blurred for anonymisation.

The annotations were performed over only the RGB stream of the robot's head camera, and not taking into account all the four video streams available from the collected data. Similarly to (Tanaka et al., 2007), the annotators were asked to indicate in real-time how engaged people interacting with a robot appeared to be in a video captured by the robot (e.g., Figure 4). They operated a dial using a game-pad joystick while watching the interaction videos using the NOVA annotation tool^2^ (Baur et al., 2015). This procedure allowed the generation of per-frame annotations of the provided videos, with very little time spent on software training (around 20 min per annotator) and on the annotation process itself (not more than the duration of the videos).

Three subsets of the overall dataset were randomly drawn and assigned to the annotators. The subsets were partially overlapping in order to enable an analysis of the inter-rater agreement for assessing the reliability of the essentially subjective metric, but also to maximize annotation coverage of the dataset. As indicated in Table 2, the total length of the annotated data was over 9 h, with 3 h 27 min of overlap between the annotators (resulting in 5 h 50 min of unique videos annotated). The amount of annotated data is depicted in Table 2. 96 unique videos were coded by the three annotators with a total of 146 videos (including repeated annotations) for a total duration of 9 h and 17 min. In total the annotated video set features 227 people [53.74% (122) females and 46.26% (105) males, 60.79% (138) adults, and 39.21% (89) minors]. The composition of each group of people interacting with the robot is very diverse; on average each videos features 2.41 people (*min* = 0, *max* = 9, σ = 1.56), 1.32 females (*min* = 0, *max* = 6, σ = 0.89), 1.14 males (*min* = 0, *max* = 5, σ = 1.26), 1.5 adults (*min* = 0, *max* = 5, σ = 0.97), and 0.96 minors (*min* = 0, *max* = 6, σ = 1.14).

**Table 2:** Video annotations by annotator (coder): unique indicates length of video coded by a single coder.

**Coder**	**# Videos**	**Tot. duration**
Coder1	66	3 h 59 min
Coder2	40	2 h 55 min
Coder3	40	2 h 23 min
Unique	94	5 h 50 min
Total	146	9 h 17 min

#### 4.2.1. Coding Evaluation

**Figure 5:**
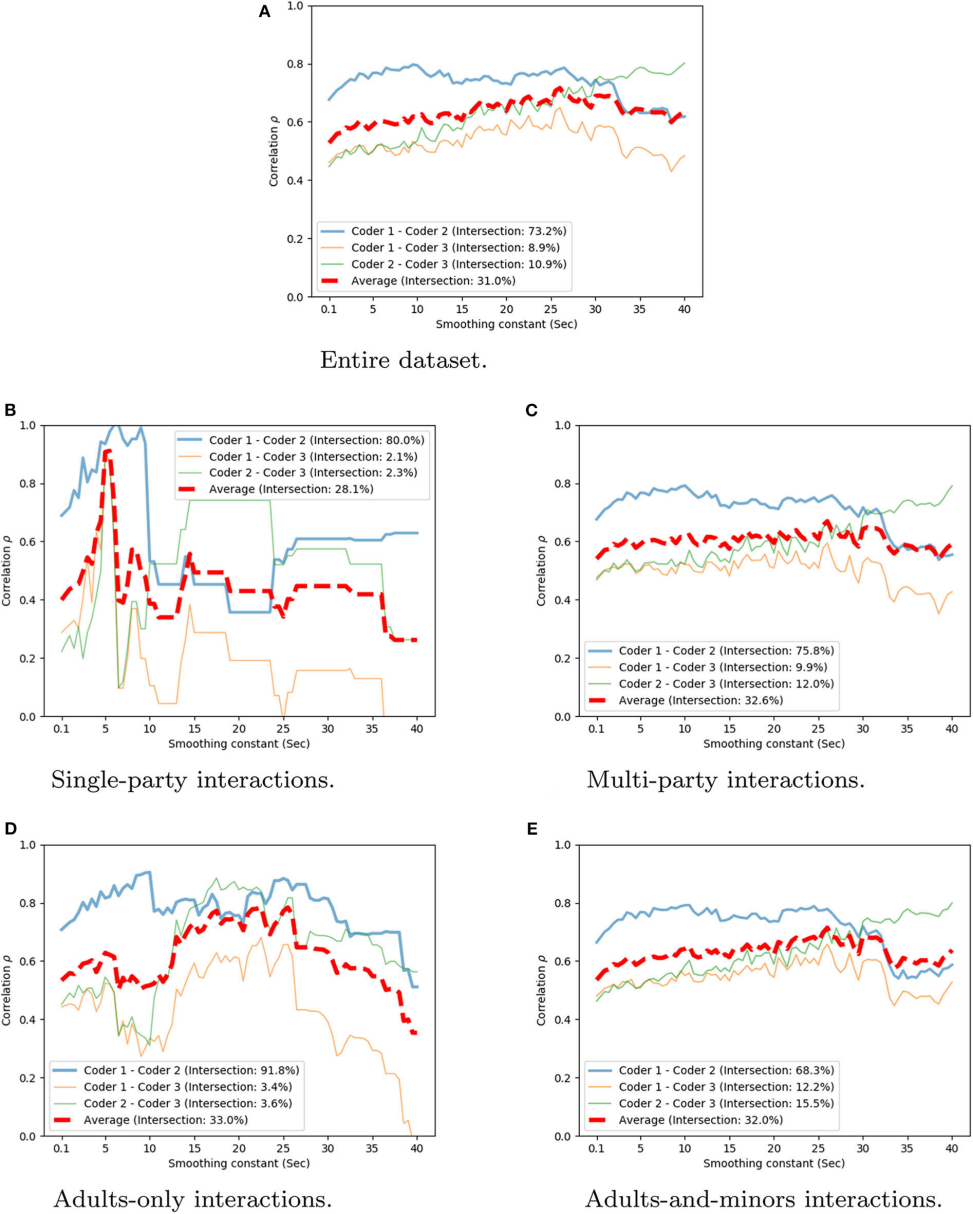
Spearman correlation averaged over coder pairs and weighted by the overlap rate. Value reported over different smoothing constants *S*. **(A)** Entire dataset. **(B)** Single-party interactions. **(C)** Multi-party interactions. **(D)** Adults-only interactions. **(E)** Adults-and-minors interactions.

The annotated engagement rating is a continuous scalar for every frame of video data. As such, Spearman's rank correlation (ρ) is employed to assess inter-rater agreement. Table 3 shows the correlation values for each pair of annotators. Since every frame is annotated (with a frame-rate of 10 frames-per-second), the continuous values were smoothed over time, using different smoothing constant values, in the range [0.1*s*, 40*s*] (Figure 5A). Table 3 provides a summary of these, with overall mean agreement rates at selected representative values of the smoothing constant. While there is some variability in the between-coder agreement, mean values of ρ vary in strength from moderate to strong (0.56 to 0.72). In this regard, there is a trade-off to be made between the smoothing constant size and the apparent agreement between the coders: the larger time window size reduces the real-time relevance of the engagement assessment, even though the agreement over the extended periods of time is greater than in comparatively shorter windows. Overall, these results indicate that the use of the independently coded data can be considered reliable in terms of the highly variable and subjective metric of engagement.

**Table 3:** Spearman's Correlation ρ at different smoothing constant values *S*.

**Coders pair**	**S [s]**	**ρ**
Coder1 ↔ Coder2	1	0.71
	5	0.77
	10	0.79
	26	0.78
Coder1 ↔ Coder3	1	0.49
	5	0.5
	10	0.52
	26	0.65
Coder2 ↔ Coder3	1	0.48
	5	0.5
	10	0.53
	26	0.72
Average	1	0.56
	5	0.59
	10	0.62
	26	0.72

*The significance p < 0.001 and sample size n ≥ 89 for all coder pairs and smoothing constants*.

Additionally, we perform an analysis targeted at an understanding of how well the annotations performed are for different interaction conditions. We separate the dataset in the 4 subsets below:

- *Single-party* interactions, i.e., with less than 2 people (22 unique videos);
- *Multi-party* interactions, i.e., with at least 2 people (72 unique videos);
- *Adults-only* interactions, i.e., where all the users are adults (38 unique videos);
- *Adults-and-minors* interactions, i.e., where there is at least 1 minor (54 unique videos).

Figures 5B–E shows the correlation value at various timescales for each of these conditions. We can observe that in the *single-party* and *adults-only* conditions the correlation is higher than in the two remaining conditions for some smoothing values. This result indicates that the assessment of engagement for group interactions and in the presence of children is affected more heavily by the differences of the individual coders. It is important to notice that the coders were not instructed to use different coding strategies for each interaction condition but to code the overall group engagement as explained above.

## 5. The Engagement Regression Model

Given the ground-truth provided by the human-coded engagement levels of user's interactions with the robot, we propose a deep learning approach for the estimation of human engagement from video sequences. The model is trained end-to-end from the raw images coming from the robot's head camera to predict a high-level engagement score of people interacting with the robot. It should be noted that this model does not model individual humans in the view of the robot but provides an overall holistic engagement score.

**Figure 6:**
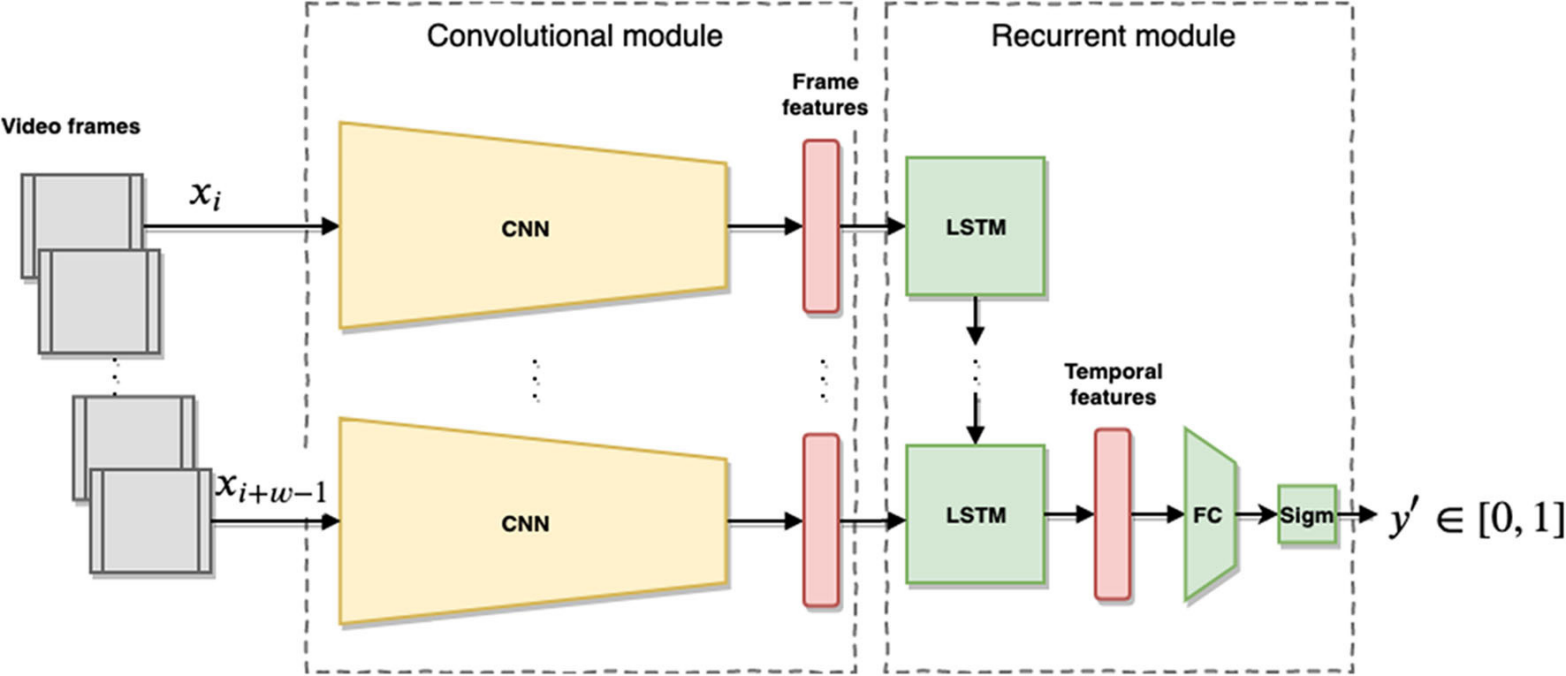
Overview of the proposed model. The input is a video stream of interactions between the robot and humans collected in *w* size intervals. The frames *x*_*i*_ are passed through the pre-trained CNN (ResNet) producing a per-frame feature vector which is then passed sequentially to the LSTM network. After *w* steps the LSTM produces a temporal feature vector which is passed to a FC layer with sigmoid activation to produce an engagement value *y* for the temporal window.

The network architecture, depicted in Figure 6, is composed of two main modules: a convolutional module which extracts frame-wise image features and a recurrent module that aggregates the frame features over a time to produce a temporal feature vector of the scene. The convolutional module is a ResNetXt-50 Convolutional Neural Network (CNN) (Xie et al., 2017) pre-trained on the ImageNet dataset (Krizhevsky et al., 2012). We obtain the frame features from the activation of the last fully connected layer of the CNN, with dimension 2048, before the softmax layer. The recurrent module is a single layer Long-Short Term Memory (LSTM) (Hochreiter and Schmidhuber, 1997) with 2048 units followed by a Fully Connected (FC) layer of size 2048 × 1. The LSTM takes in input a sequence of *w* frame features coming from the convolutional module and produces, in turn, a feature vector that represents the entire frame sequence, to capture the temporal behavior of humans within the time window *w*. The temporal features are passed through the FC layer with a sigmoid activation function at the end to produce values *y*′ ∈ [0, 1] The recurrent module is trained in our experiments to predict engagement values from the provided annotation values, while the CNN layer is fixed.

The proposed framework is implemented in Python using the Keras library (Chollet, 2015) and is freely released as a ready-to-use ROS package to the HRI community^3^.

## 6. Experiments

We train and test the model presented in section (5) on our own TOGURO dataset, and assess generalization of this model (without modification) on the public UE-HRI dataset in the following subsections.

### 6.1. TOGURO Dataset Processing

We used the entire annotated dataset presented in section (4.2), composed of 94 videos, for a total duration of 5 h and 50 min of interactions. For each video, we randomly choose an annotation if multiple are available from the different coders (see Table 2), in order to avoid repetitions in the data and biasing the model toward those videos that have been annotated multiple times. Each video is then randomly assigned to either the training, test or validation set with a corresponding probability of 50s, 30, and 20%, respectively, to prevent our model to train and test over data that are closely correlated at the video frames level. Sampling for the dataset split hence operates on full video level, rather than on frame level. Each video *V*_*k*_ is composed of *I*_*V*_*k*__ frames *x*_*i*_ ∈ *V*_*k*_ for *i* ∈ 0, …, *I*_*V*_*k*__ and has an associated array of annotations *A*_*k*_ = [*y*_0_, …, *y*_*I*__*V*__*k*___], also of dimension *I*_*V*_*k*__. From all the videos in each set (training/test/validation) we extract all the possible sequences of *w* consecutive frames *X*_*i*_ = [*x*_*i*_, …, *x*_*i*+*w*−1_] to be the input sample for our model. Therefore, each sample *X*_*i*_ has an overlap of *w* − 1 frames with the consecutive sample *X*_*i*+1_ from the same video. For each sample *X*_*i*_ we assign the ground truth value *y*_*i*+*w*−1_ ∈ *A*_*k*_, in order to relate each sequence of frames with the engagement value set at the end of the sequence.

After the pre-processing phase over our dataset, we obtain 93,271 training samples, 72,146 test samples and 44,581 validation samples. Each frame is reshaped to 224 × 224 pixel frames, and normalized before being fed to the network.

### 6.2. Training and Evaluation

For training and evaluation, we decided to set the window size *w* equal to 10 frames in order to have a model that gives evaluations of the engagement in a relatively short time (i.e., after 1 s). Even though more temporally extended time windows would provide more coherent ground truth values among the different annotators, as discussed in section (4.2.1), we decide to sacrifice some accuracy in favor of increase real timeless of our mode predictions.

During training the weights of the Convolutional module, which is already pre-trained, are kept frozen while the Recurrent module is fully trained from scratch. The model is trained to optimize the Mean Squared Error (MSE) regression loss between the prediction values $y_{i}^{\prime }$ and the corresponding ground truth values *y*_*i*_ using the Adagrad optimization algorithm (Duchi et al., 2011) with an initial learning rate *lr* = 1*e* − 4. At each training epoch, we sample uniformly 20% of the training set samples to be used for training and we collect them in batches of size *bs* = 16. The uniform data sampling of the training data is performed in order to reduce training time and limiting overfitting (El Korchi and Ghanou, 2019). The model has been trained for a total of 22 epochs using early stopping after no improvement in validation loss.

The evaluation of the model is performed on the entire test set, which is composed of samples from videos never encountered in the training set. Additionally, we evaluate the performances of the model on the *single-party, multi-party, adults-only* and *adults-and-minors* portions of the test set separately to understand how this is affected by the differences in the group of users. In this last experiment, we continue to use the model learned from the entire training set and not from a different portion of the training set for each condition.

### 6.3. Assessing Generalization

In order to assess the generalization capabilities of our trained model over different scenarios featuring people interacting with robots, we propose to test the performance of our trained model as a detector of the start and end of interactions over the UE-HRI dataset (Ben-Youssef et al., 2017).

#### 6.3.1. The UE-HRI Dataset

Similarly to our dataset, the UE-HRI dataset has been collected during a public deployment of a social robot (i.e., SoftBank Robotics' Pepper) featuring interactions in-the-wild with groups of users. Also, the human-robot interactions are mediated through a touchscreen interface and speech. However, while our robot moves around the museum together with the users as it explains the various exhibits, the Pepper robot stands in a fixed position in the room and interacts with people that enter its own engagement zone by asking them questions and showing applications from the touchscreen.

The dataset provides videos of the interactions from the robot's head cameras. Even though the cameras move together with the Pepper's head when the robot shifts its gaze from one person to another, their position allows to estimate the user's engagement from the robot's point of view. The videos are accompanied by annotations of start/end of interactions and various signs of engagement decrease [Sign of Engagement Decrease (SED), Early sign of future engagement BreakDown (EBD), engagement BreakDown (BD) and Temporary Disengagement (TD)]. These annotated signals are associated with cues of verbal/non-verbal behaviors of the users and other various features, like the users' position. In total, the dataset features 54 interactions with 36 males and 18 females, where 32 are mono-users and 22 are multiparty.

#### 6.3.2. Evaluation Procedure

For a fair comparison with our proposed method, we evaluate the ability of our model to distinguish between the moments during which an interaction is taking place and those in which there is a breakdown (TD or BD), the interaction is not yet started or it is already ended, in line with the UE-HRI coding scheme. Consequently, we predict engagement values over the RGB image streams from the Pepper robot's front camera. By setting a threshold value *thr* we convert the predictions *y*′ into a binary classification of *C* = {⊤, ⊥} (prediction above or below *thr*) which indicates whether there is engagement or not. The categorical predictions are then compared with values from the annotations in the dataset. We consider the ground truth value to be $y_{int}^{t}=⊤$ if at time *t* there is an annotation of a *Mono* or *Multi* interaction and there are no annotations of BD or TB in the UE-HRI coding. The ground truth value is $y_{int}^{t}=⊥$ otherwise.

## 7. Results

With our evaluation, we set out to provide evidence that our model can predict engagement through regression on our own TOGURO dataset by assessing its accuracy in comparison to the ground-truth annotation, and to assess the generalization ability of the model on newly encountered situations through the analysis of the UE-HRI data.

To show the ability of our framework to map short-term human behavioral features from image sequences into engagement scores, we compute the Mean Squared Error (MSE) prediction loss on our test set as 0.126 (in the context of the [0, 1] interval of output expected), also reported in Table 4. Additionally, we report the Spearman's rank correlation ρ between our model's predictions and the ground truth values of our model showing that it is consistent with the inter-rater agreement results reported in section (4.2.1). The results of evaluating the trained model on the four different conditions of the group of users shows that the model is able to predict the engagement more accurately in the *single-party* and *adult-only* conditions, reported in Table 5. This also is in line with the inter-rater agreement results for the conditions in section (4.2.1).

**Table 4:** Model performance on our TOGURO Dataset.

**Test loss (MSE)**	**Correlation ρ**	**Prediction time**	**Memory usage**
0.126	0.634	*t* <= 0.2 s	5.4 GB

*ρ measures the correlation between the predictions and the ground truth values with smoothing factor S = 1 [s]. Prediction time is relative to the GPU GeForce GTX 1060 we have used to carry out the assessment*.

**Table 5:** Model performances on different conditions of users group composition in terms of MSE test loss and Spearman's Correlation ρ [with smoothing factor *S* = 1 s] of predictions with the ground truth values.

**Condition**	**Test loss (MSE)**	**Correlation ρ**
Single-party	0.087	0.758
Multi-party	0.136	0.622
Adults-only	0.068	0.812
Adults-and-minors	0.149	0.563

Looking back at section (1), a soft real-time operation is seen as a requirement for the applicability of our model. Hence, we measured the duration of a forward pass on our GPU hardware of 10 consecutive frames (1 sample) through the convolutional module and the recurrent module taking at most 200*ms*, allowing real-time estimation of engagement at 5 frames per second.

**Figure 7:**
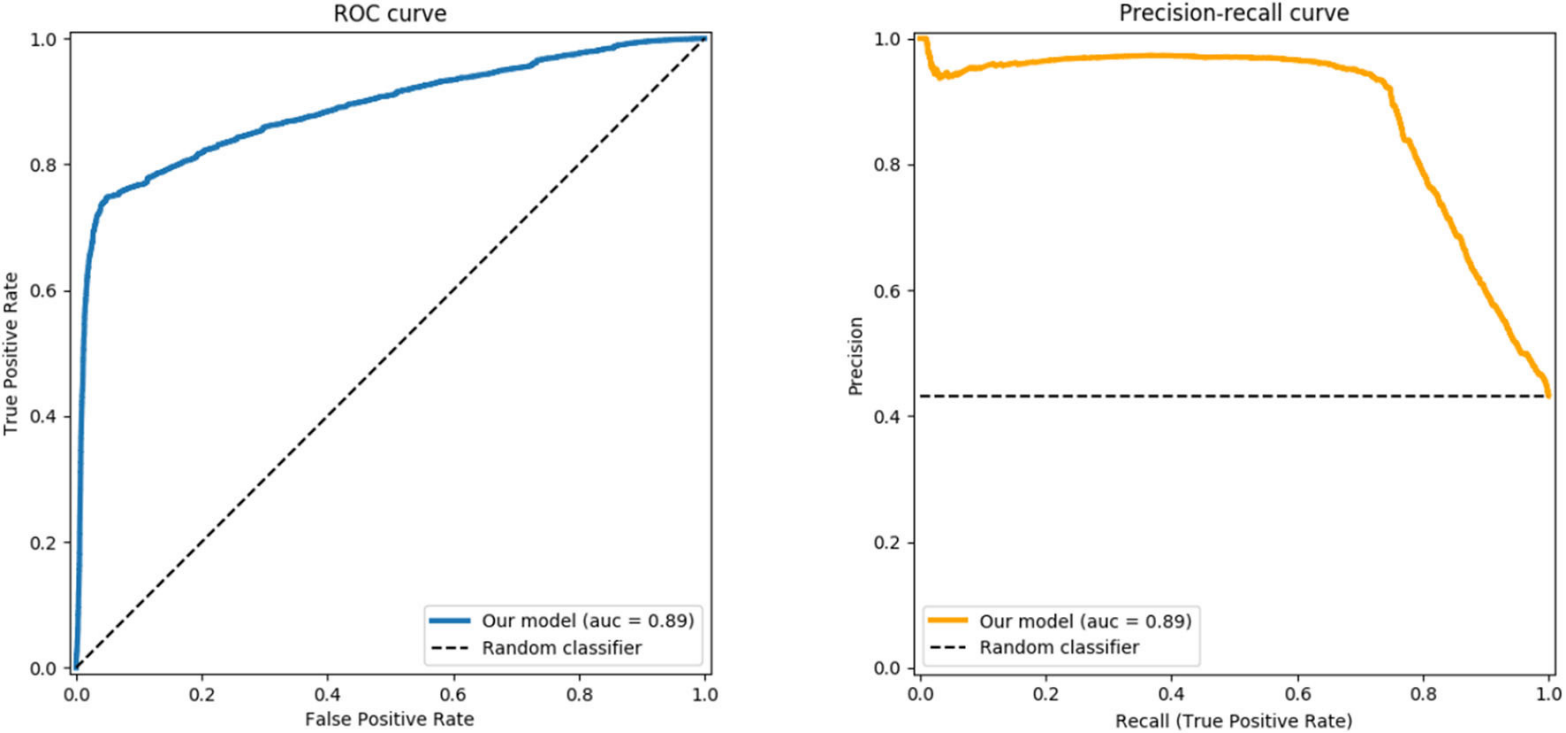
ROC curve **(left)** and Precision-Recall curve **(right)** generated using our trained model as a classifier of the interaction sessions for the UE-HRI dataset.

Evaluating the power of our approach for binary classification on the UE-HRI as detailed above in section (6.3), allows us to capture the generalization capabilities. In Figure 7, we report the Receiver Operating Characteristic (ROC) and the Precision-Recall curves obtained by varying the threshold with values in the range *thr* ∈ [0, 1] of the binary classification task on the UE-HRI data. The Area Under the Curve (*AUC* = 0.89 in our experiment) reports the probability that our classifier ranks a randomly chosen positive instance $y_{int}^{t}=⊤$ higher than a randomly chosen negative one $y_{int}^{t}=⊥$, i.e., provides a good assessment of the performance of the model in this completely different dataset.

**Figure 8:**
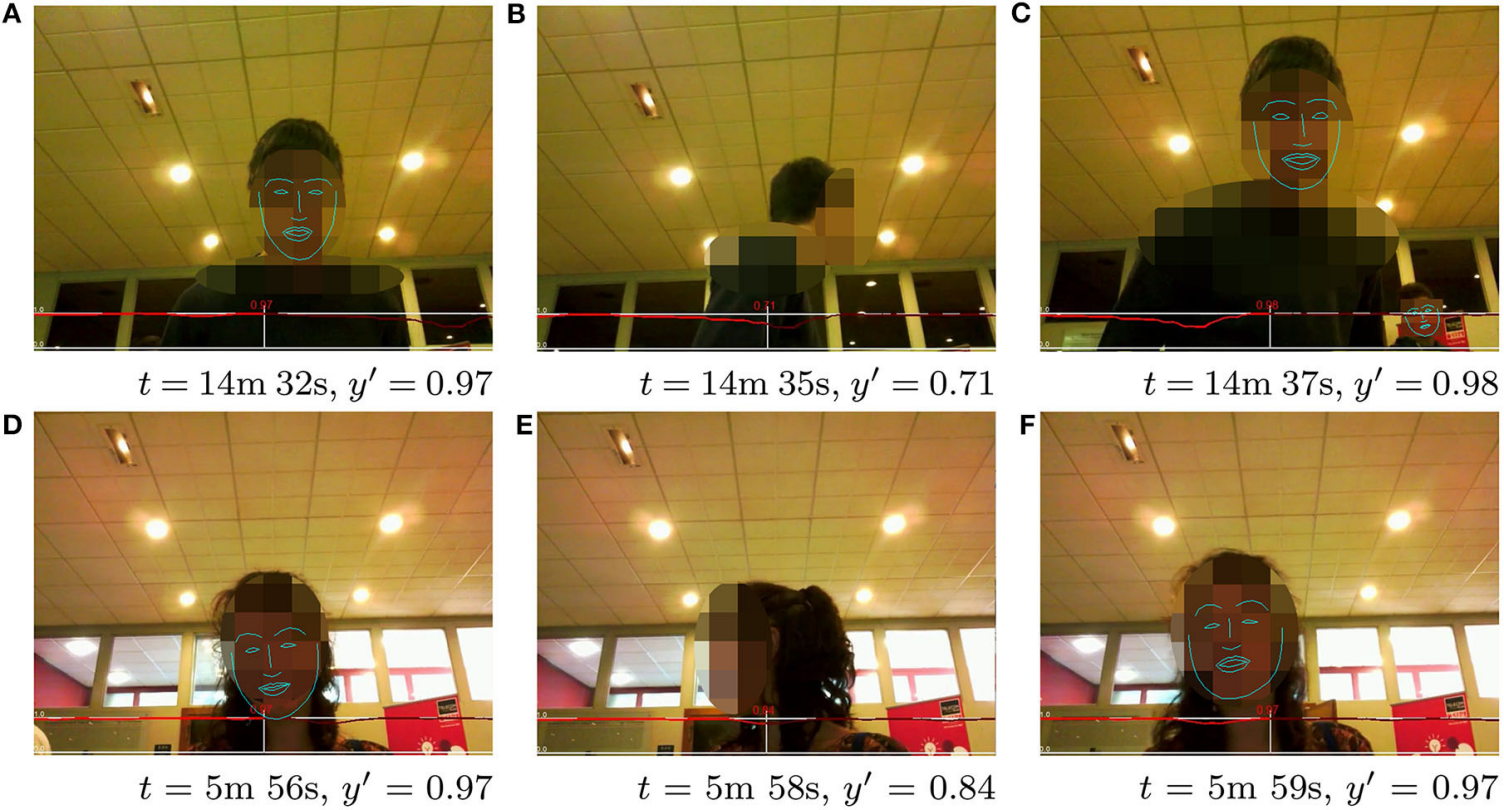
**(A-F)** UE-HRI dataset: two sequences of short timescale sequential frames showing how the temporal diverting of attention is reflected in the model predicting a lower engagement value. The red plot shows the predicted engagement values over the frame sequences, with the prediction *y*′ at the frame shown in the picture at time *t* being in the center, past predictions on the left and future predictions on the right. Faces from original dataset blurred for anonymisation; face landmarks added in postprocessing to indicate face orientation. Permission for re-use of the images has been obtained from the copyright holder.

**Figure 9:**
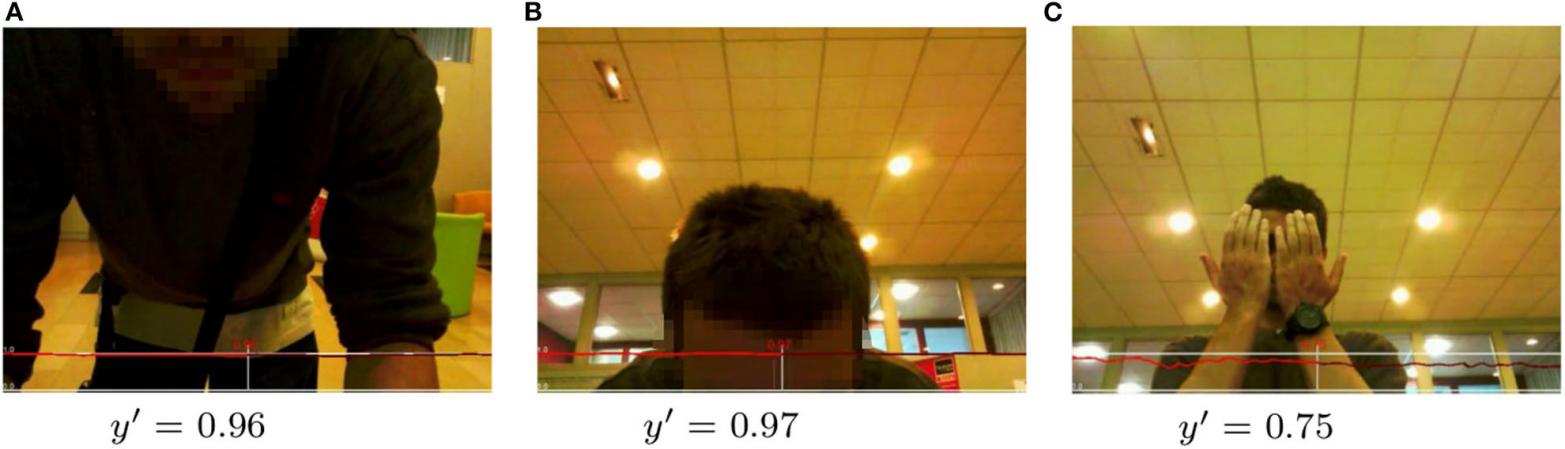
**(A-C)** UE-HRI dataset: examples of correct prediction of high engagement (*y*′ >= 0.75) in situations difficult to understand using standard face description features. The red plot shows the predicted engagement values over the frame sequences with the prediction *y*′ at the frame shown in the picture being in the center, past predictions on the left and future predictions on the right. Faces from original dataset blurred for anonymisation. Permission for re-use of the images has been obtained from the copyright holder.

**Figure 10:**
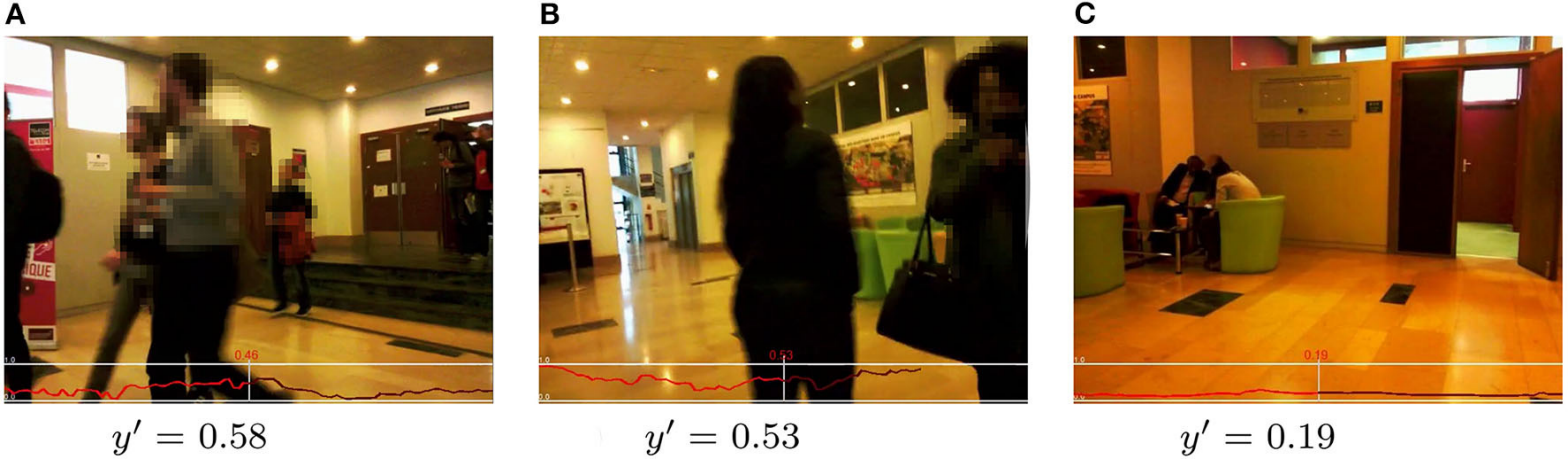
**(A-C)** UE-HRI dataset: examples of correct low/medium engagement prediction (*y*′ <= 0.6) in cases in which the people were not actually engaging with the robot. The red plot shows the predicted engagement values over the frame sequences with the prediction *y*′ at the frame shown in the picture being in the center, past predictions on the left and future predictions on the right. Faces from original dataset blurred for anonymisation. Permission for re-use of the images has been obtained from the copyright holder.

Given these encouraging quantitative results, some qualitative assessments of exemplary frames with the corresponding computed engagement score are presented in Figures 8–10. All figures show examples of the UE-HRI dataset, which was completely absent from the training dataset (section 6.1). Figure 8 presents two short sequences (roughly 2 s apart between frames), showcasing short-term diversion of attention of subjects resulting in a temporarily lower engagement score, but not leading to a very low engagement level. Figure 9 exemplifies that our model can cope well with perception challenges which would forgo a correct assessment just using gaze or facial feature analysis. While one could, in this context, argue that our model has simply learned to detect people, Figure 10 is providing three examples from different videos of the UE-HRI dataset with people present in the vicinity of the robot, but not engaging with it. The engagement scores in these examples are significantly lower across all frames.

These qualitative reflections are evidently supported by the quantitative analysis of both datasets, providing us with confidence that the trained model is broadly applicable and can serve as a very useful tool to the HRI community with its modest computational requirements and high response speed in assessing videos from a robot's point of view.

## 8. Discussion and Conclusion

This paper has motivated, developed and validated a novel easy-to-use computational model to assess engagement from a robot's perspective. The results presented in the previous sections lead us to the conclusion that:

1. A moderate to strong inter-rater agreement (see Table 3) in measuring engagement on [0, 1] interval indicates that humans can reasonably and reliably assess the holistic engagement from a robot's point of view solely from video;
2. A two-stage deep-learning architecture as presented in Figure 6 trained from our TOGURO dataset is a suitable computational regression model to capture the inherent human interpretation of engagement provided by the annotators; and that
3. The trained model is generic enough to be successfully applied in a completely different scenario, here the UE-HRI dataset, showing the applicability of the model also in different environments, on a different robot with a different camera, and with different tasks and people. The area under the Receiver-Operator Curve (ROC) of 0.89 and the Precision-Recall curve in Figure 7 provide evidence that indeed the proposed regression model can serve as a strong discriminator to identify situations of loss of engagement (TD or BD in the UE-HRI coding scheme).

Our results confirm the idea that the human holistic assessment of an abstract quantity, like the engagement, can be utilized as a coherent metric for learning a prediction model of that same quantity. While a cue-centric model based on specific perceptual features, such as gaze, can be more easily interpretable, it can miss out on important events that are not explained by the chosen features. A model learned from raw data, like the one presented in this study, can instead learn to recognize what are the important features to take into account for the assessment.

We hypothesize that the learned model does not solely discriminate person and/or face presence, but that the temporal aspects of the human behavior observable in the video are captured by the LSTM layer in our architecture well enough to successfully deal with these situations. The correlation values between the model predictions and the ground truth value in Tables 4, 5, suggest that the predictions are in line with the coders' assessment, even when averaging at a short timescale like 1 s. This is important because it means that the model can be used to immediately identify moments of decreased engagement and plan to recover from it before the users completely disengage with the robot. As the next step in that direction, we plan to use reinforcement learning (RL) techniques by integrating our engagement model predictions as part of the reward function given to the robot. As typically done in RL problems, the reward function provides only a scalar value at each time step to the agent to learn from, even though the function itself can be a combination of different objectives we want the agent to optimize. Therefore, our engagement scalar values can be seamlessly integrated into our robot reward function.

## Data Availability Statement

The datasets generated for this study will not be made publicly available as the ethical approval does not allow the public release of any data that can feature identifiable persons.

## Ethics Statement

The studies involving human participants were reviewed and approved by University of Lincoln's Ethics Board. Written informed consent from the participants or the participants' legal guardian/next of kin was not required to participate in this study in accordance with the national legislation and the institutional requirements.

## Author Contributions

All authors contributed to the conception and design of the study, and wrote sections of the manuscript. FD performed the collection of the dataset, implementation and training of the model, and analysis of the results. All authors contributed to manuscript revision, read, and approved the submitted version.

## Conflict of Interest

The authors declare that the research was conducted in the absence of any commercial or financial relationships that could be construed as a potential conflict of interest.

## Footnotes

^1^https://www.thecollectionmuseum.com/robot-at-the-collection

^2^https://github.com/hcmlab/nova

^3^https://github.com/LCAS/engagement_detector
